# Optimizing Dacarbazine Therapy: Design of a Laser-Triggered Delivery System Based on β-Cyclodextrin and Plasmonic Gold Nanoparticles

**DOI:** 10.3390/pharmaceutics15020458

**Published:** 2023-01-30

**Authors:** Sebastián Quintana-Contardo, Orlando Donoso-González, Erika Lang, Ariel R. Guerrero, Michael Noyong, Ulrich Simon, Marcelo J. Kogan, Nicolás Yutronic, Rodrigo Sierpe

**Affiliations:** 1 Departamento de Química, Facultad de Ciencias, Universidad de Chile, Las Palmeras 3425, Ñuñoa, Santiago 7800003, Chile; 2 Departamento de Química Farmacológica y Toxicológica, Facultad de Ciencias Químicas y Farmacéuticas, Universidad de Chile, Santos Dumont 964, Independencia, Santiago 8380494, Chile; 3 Advanced Center for Chronic Diseases (ACCDiS), Universidad de Chile and Pontificia Universidad Católica de Chile, Santiago 8380494, Chile; 4 Institute of Inorganic Chemistry, RWTH Aachen University, Landoltweg 1a, 52074 Aachen, Germany; 5 Departamento de Química, Facultad de Ciencias Naturales, Matemática y del Medio Ambiente, Universidad Tecnológica Metropolitana (UTEM), Las Palmeras 3360, Ñuñoa, Santiago 7800003, Chile

**Keywords:** dacarbazine, β-cyclodextrin, gold nanoparticle, inclusion complex, drug delivery, laser irradiation, triggered drug release

## Abstract

Dacarbazine (DB) is an antineoplastic drug extensively used in cancer therapy. However, present limitations on its performance are related to its low solubility, instability, and non-specificity. To overcome these drawbacks, DB was included in β-cyclodextrin (βCD), which increased its aqueous solubility and stability. This new βCD@DB complex has been associated with plasmonic gold nanoparticles (AuNPs), and polyethylene glycol (PEG) has been added in the process to increase the colloidal stability and biocompatibility. Different techniques revealed that DB allows for a dynamic inclusion into βCD, with an association constant of 80 M^−1^ and a degree of solubilization of 0.023, where βCD showed a loading capacity of 16%. The partial exposure of the NH_2_ group in the included DB allows its interaction with AuNPs, with a loading efficiency of 99%. The PEG-AuNPs-βCD@DB nanosystem exhibits an optical plasmonic absorption at 525 nm, a surface charge of −29 mV, and an average size of 12 nm. Finally, laser irradiation assays showed that DB can be released from this platform in a controlled manner over time, reaching a concentration of 56 μg/mL (43% of the initially loaded amount), which, added to the previous data, validates its potential for drug delivery applications. Therefore, the novel nanosystem based on βCD, AuNPs, and PEG is a promising candidate as a new nanocarrier for DB.

## 1. Introduction

Aging and exposure to pollutants make cancer one of the leading causes of death in the world, with 10 million deaths occurring in 2020 and projected to reach 28 million until 2040 [1,2]. Antineoplastic drugs have mechanisms of action based on interrupting cell growth or destroying them; they affect both cancerous and healthy cells [3,4]. Dacarbazine (DB) is an alkylating agent used in the treatment of several types of cancer, such as Hodking’s lymphoma, melanoma, pancreatic islet carcinoma, and sarcomas [4,5,6,7]. It has an aggressive and non-selective behavior and is classified among the antitumor drugs for which there is no exposure range considered safe; nevertheless, its consumption has increased by around 12% annually since 2004 [8,9]. The drug is highly unstable, degrading to carbene and aryl radicals, which are species involved in DNA damage, and inside the human body it produces reactive and highly toxic oxygen species. These species are also suspected to be responsible for the pain experienced by patients and for the incidence of carcinogenic, teratogenic, and mutagenic effects [10,11]. This antineoplastic has a 5-h half-life in the body and a 40% intact excretion rate in urine. Even from an ecological point of view, after biodegradation processes in wastewater treatment plants, 98.2% remains, generating a concentration of 0.1 ng/L in riverbeds, which is a serious environmental problem. [12,13]. Therefore, it is necessary to further advance the application of this drug, for example, by improving its aqueous solubility, stability, and specificity for an intelligent therapy at the site of action, ideally reducing the administered dose. In this sense, cyclodextrins (CD) and gold nanoparticles (AuNPs) have emerged as promising tools for the development of novel drug delivery nanosystems [2,14,15,16,17].

CDs are nontoxic cyclic oligosaccharides formed from 6, 7, or 8 glucopyranose units bonded via α(1−4) bonds as a truncated cone, approved by the Food and Drug Administration (FDA) for a wide range of pharmaceutical, food product, and cosmetic applications [18,19,20]. They are characterized by a hydrophilic exterior and a hydrophobic interior, working as a pocket or host for poorly soluble or reactive organic molecules, forming inclusion complexes [21,22]. The βCD, composed of 7 glucose units, is highlighted due to its appropriate dimensions to include aromatic cycle species. Hence, it has been widely studied to optimize the performance of drugs by preventing decomposition and increasing aqueous solubility [23,24]. Considering the molecular dimensions of DB and its hydrophobic regions, this drug could be included inside the non-polar internal cavity of βCD, forming a new inclusion complex [25,26]. Interestingly, some inclusion complexes present an arrangement that exposes suitable drug functional groups to interact with other materials, such as AuNPs, to add characteristics and properties that continue to contribute to the optimization of the drug in therapy [27,28,29,30,31].

AuNPs have been extensively studied in therapy and the diagnosis of cancer [2,32,33,34]. The extensive study on chemical synthesis methods allowed a high degree of control over the size and shape of the nanosystem obtained, resulting in simple, reproducible, and scalable methodologies [35,36,37]. These nanoparticles demonstrate excellent biocompatibility, interesting optoelectronic properties, and an easily functionalized surface, which leads to a promising platform for drug delivery [38,39,40,41]. The use of polymers and proteins, such as polyethylene glycol (PEG) and bovine serum albumin (BSA), are used to increase their stability and avoid opsonization of AuNPs, thus increasing their circulation time in the organism [42,43]. Due to their size and the size of blood vessels in fenestrated tissue, AuNPs accumulate preferentially in tumors due to passive targeting enabled by the enhanced permeation and retention effect (EPR) [44,45,46]. In this way, antineoplastic agents can be targeted to their specific site of action, minimizing damage to healthy tissue. Furthermore, drugs can be released in a controlled process utilizing the photothermal effect, which is the generation of local heat on the surface of the plasmonic AuNP due to their interaction with light at wavelengths close to the optical absorption maximum [47,48,49,50]. Currently, AuNPs are independently used as a therapeutic agent by triggering hyperthermia to destroy tumor cells [51]. Thus, the vectorization of DB on AuNPs could optimize its toxic profile and synergize at the therapeutic level, while the solubility and stability in water could be increased by formulating it in βCD, which allows the formation of a new nanosystem for site-specific and controlled release [51,52,53,54].

**Figure 1:**
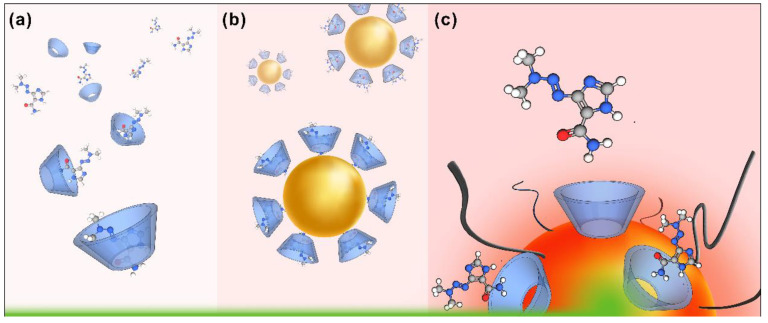
Schematic representation of the development of the PEG-AuNPs-CD@DB nanosystem: (**a**) formation of the CD@DB complex by drug inclusion into the cyclodextrin cavity, (**b**) coating of the AuNPs surface with the CD@DB complexes, and (**c**) release of the DB from the nanosystem triggered by laser irradiation.

This is the first study of the inclusion of DB in βCD in a solid state, and its subsequent functionalization with AuNPs. Considering the exclusive use of biocompatible agents, PEG was incorporated into the colloidal solution, forming the new PEG-AuNPs-βCD@DB nanosystem. The understanding of the complex formation was performed using techniques including powder X-ray diffraction, NMR of 1 and 2 dimensions, scanning electron microscopy, and molecular docking. The development of the nanosystem was characterized using UV-Visible spectroscopy, DLS, Zeta potential measurements, Raman and SERS spectroscopy, and transmission electron microscopy. Validation of this nanosystem as a new nanocarrier candidate was performed by obtaining relevant pharmaceutical parameters such as the association constant, loading capacity and efficiency, and size and surface charge of functionalized nanoparticles. As a proof of concept, the release of DB was demonstrated by laser irradiation assays. Figure 1 shows a scheme of the different stages of the nanosystem’s development and its proof of concept.

We believe that the synergistic incorporation of βCD, AuNPs, and PEG could improve the formulation of DB-optimized anticancer therapy by increasing the aqueous solubility, stability, and local concentration of the drug, in addition to triggering its release at the site of action by laser irradiation.

## 2. Materials and Methods

### 2.1. Materials

Dacarbazine (C_6_H_10_N_6_O) ≥ 99%, molar weight: 182.18 g/mol; β-cyclodextrin (C_42_H_70_O_35_) ≥ 97%, molar weight: 1134.98 g/mol; tetrachloroauric acid (HAuCl_4_*3H_2_O) ≥ 99.9%, molar weight: 393.83 g/mol; sodium citrate (Na_3_C_6_H_5_O_7_) ≥ 99%, molar weight: 294.10 g/mol; and nitric acid (HNO_3_) 70%, molar weight: 63.01 g/mol were provided by Sigma Aldrich (Saint Louis, MO, USA). Hydrochloric acid (HCl) 37%, molar weight: 36.46 g/mol; ethanol (C_2_H_5_OH) ≥ 99.9%, molar weight: 46.07 g/mol; hexane (C_6_H_14_) ≥ 99.7%, molar weight: 86.18 g/mol; and water (nanopure) were provided by Merck (Darmstadt, Germany). Methoxy PEG Thiol (CH_3_O-PEG5000-SH) ≥ 95%, molar weight: 5 kDa was provided by JenKem Technology (Plano, TX, USA).

### 2.2. Formation of the Inclusion Complex

To form the βCD@DB complex, the saturated solutions of method [55] were used with minor modifications. A total of 96 mg of DB were dissolved in a 1:2 water ethanol solution with constant stirring at 25 °C for 1 h. In addition, 400 mg of βCD were dissolved in water with constant stirring for 2 h at room temperature. Then, both solutions were mixed with gentle and constant stirring for 1.5 h. The mixture was covered and kept motionless under a hood for 2 weeks. Crystals precipitated at the bottom of the solution and were extracted, washed, and dried before the complete evaporation of the solvents.

### 2.3. Loading Capacity of β-Cyclodextrin

Loading capacity was calculated from the weights of βCD and drug obtained, using Equation (1) [56].

(1) $$Loading\;capacity=\frac{Weight\;of\;DB\;in\;\beta CD}{Weight\;of\;\beta CD}\times 100$$

### 2.4. Association Constant of β-Cyclodextrin@dacarbazine

For the βCD@DB complex, the association constant was obtained following the Higuchi and Connors method [57]. The βCD concentration versus the loaded DB concentration (calculated by the Beer–Lambert law) was plotted. The value of the slope of the graphs related the amount of βCD added to the amount of solubilized drug, indicating the degree of solubilization (DS). DS was used in Equations (2) and (3) to finally calculate the association constant (*Ka*_1:1_) and complexation efficiency (CE) of the βCD@DB.

(2) $$Ka_{\left(1:1\right)}=\frac{\mathrm{DS}}{\left[\mathrm{Co}\right]\left(1-\mathrm{DS}\right)}$$

(3) $$\mathrm{CE}=Ka_{\left(1:1\right)}\left[\mathrm{Co}\right]=\frac{\mathrm{DS}}{\left(1-\mathrm{DS}\right)}$$

[Co] corresponds to the concentration of the free drug in absence of βCD.

### 2.5. Stability and Modeling of β-Cyclodextrin@dacarbazine

Conformational searches were calculated by Autodock Vina 1.1.2 software (2014) [58] (Lo Jolla, CA, USA) with the Lamarckian Genetic Algorithm (LGA), using βCD as the receptor and DB as the ligand. The structure of βCD was extracted from the crystallographic data reported in the Cambridge Crystallographic Database Nº 210,892 [59]. The parameters used for the global search were consistent with previous similar works [27,60,61], where an initial 300 individuals, 2000 maximum energy evaluations, and 10,000 maximum generations were used as a final criterion. An elitism value of 1 mutation rate of 0.02 and a crossover rate of 0.8 were used. The vacuum permittivity was set up. The amine groups were considered rotable, thus producing the flexible docking scheme. The modeling grid measures were 4 × 4 × 4 nm to cover the entire surface of βCD, which calculated the most favorable complexation positions for the system. The obtained structures of the βCD@DB complex were employed to rationalize the experimental results.

### 2.6. Synthesis of Gold Nanoparticles

A synthesis of plasmonic AuNPs was performed using the Turkevich method [62]. All glassware were carefully washed with aqua regia before use. A reflux system was used, and 100 mL of HAuCl_4_*3H_2_O 1.0 mM was added. An amount of 114.1 mg of sodium citrate was dissolved in 10 mL of water and heated at 60 °C for approximately 5 min. When reflux was achieved, a citrate solution (at 60 °C) was added under constant agitation (72 g) over 30 min. Later, the solution was cooled slowly to room temperature. The obtained AuNPs were filtered and stored at 4 °C. The concentration of AuNPs was obtained using the reported molar extinction coefficient and the Beer–Lambert equation [63,64].

### 2.7. Functionalization of Gold Nanoparticle with the Complex

AuNPs were functionalized with the βCD@DB complex via the exchange of citrate, which was the original stabilizer. In addition, thiolated polyethylene glycol (CH_3_O-PEG-SH) was incorporated. For this, 2 mL of a 20 mg/mL aqueous solution of βCD@DB were mixed with 2.5 mL of a 9 nM aqueous solution of AuNPs and agitated for 10 min at room temperature, completely protected from light sources. Subsequently, 500 μL of a 0.420 mM aqueous solution of CH_3_O-PEG-SH were added and agitated for 1 h under the same conditions. The PEG-AuNPs-βCD@DB solution that formed was dialyzed to facilitate the removal of excess citrate. For this purpose, a Spectrum™ brand cellulose exchange membrane with a pore size of 5000 d and solutions of decreasing citrate concentration down to pure water were used. The process took 2 days. Finally, the resulting colloidal solution was centrifuged at 3200 rpm and resuspended in water fixed at pH 6.5. The AuNPs-βCD@DB-PEG solution was stored at 4 °C.

### 2.8. Loading Efficiency of Gold Nanoparticles

Loading efficiency is the maximum amount of drug that the nanosystem can carry. To calculate it, the amount of DB detected in the supernatant after functionalizing the AuNPs with the βCD@DB complex was subtracted from the amount of DB initially available, using Equation (4) [56].

(4) $$Loading\;efficiency=\frac{Weight\;of\;DB\;in\;\beta CD@DB\;loaded\;to\;the\;AuNP}{Weight\;of\;DB\;in\;\beta CD@DB\;added}\times 100$$

### 2.9. Equipment and Methodology for the Characterization

A crystallographic analysis was performed by the powder X-ray diffraction technique (PXRD) using a Siemens D-5000 diffractometer (Munich, Germany) with graphite-monochromated Cu K-α radiation at 40 kV and 30 mA with a wavelength of 1.540598 Å.

SEM micrographs were obtained using a LEO 1420 VP (Oberkochen, Germany) with a coupled energy dispersive analysis instrument, model Oxford 7424, at an accelerating voltage of 25 kV. For FE-SEM imaging, a Zeiss Leo Supra 35-VP model (Oberkochen, Germany) was used with accelerating voltages of 15 kV and 20 kV. A coating with gold using magnetron sputtering in high vacuum was necessary to improve the conductivity of the samples and avoid their polarization. For βCD and DB, 100s of metallization were performed and for βCD@DB, 20s of metallization were performed.

Nuclear magnetic resonance of 1 and 2 dimensions (^1^H and ROESY, respectively) was performed using Bruker Advance 400 MHz equipment (Billerica, MA, USA) at 300 K, with TMS as the internal reference and DMSO-d_6_ as solvent. Specifically, the ROESY technique was performed using the pulsed field selected gradient method for 24 h. The processing of the data obtained was performed using the MestRec software (Santiago de Compostela, Spain).

For UV-visible spectrophotometry measurements, PerkinElmer Lambda 25 UV equipment (Waltham, MA, USA) was used with 1-cm-diameter quartz cuvettes in a wavelength range between 200 and 800 nm. 

To determine the average particle size and its distribution, high-resolution transmission electron microscopy (HRTEM) was performed using a JEOL 2000FX TEM microscope (Akishima, Tokyo, Japan) at 200 kV, and transmission electron microscopy (TEM) was performed using a JEOL JEM 1200 EX (Akishima, Tokyo, Japan) with an accelerating voltage of 80 kV. The samples were prepared by depositing 10 μL of solution on a copper grid with a continuous formvar film and were slowly dried by exposure from an IR lamp.

Dynamic light scattering (DLS) and Zeta potential techniques were performed using Malvern ZetaSizer Nano ZS equipment (Malvern, Malvern, United Kingdom) with a capillary cell with gold electrodes. 

Raman spectra were obtained from the direct measurement of the samples in the solid and aqueous states, using a Renishaw Ramanscope 1000 (Wotton-under-Edge, United Kingdom), excited with a 632.8 nm He-Ne laser. IR spectra were obtained using a Perkin Elmer 2000 (Waltham, MA, USA) with KBr pills. To interpret the results of the IR and Raman spectra, calculations with the PhEA molecule were performed using Gaussian 09, revision D.01 [65], using density functional theory at the B3LYP/6- 311 + G(d,p) level of theory in the gaseous state. Geometry optimization and frequency calculations, including Raman activity, were requested. 

### 2.10. Drug Release Studies Using Laser Irradiation

The laser used was provided by Power Technology, Inc. The wavelength was 532 nm, with a beam diameter of 1 mm and 45 mW of power. In a quartz cuvette, an aqueous phase and an organic phase were disposed of, adding 300 μL of hexane and 300 μL of the PEG-AuNPs-βCD@DB nanosystem in water, isolated from external light sources. First, the passive diffusion of the drug from the nanosystem into the organic phase was studied. Then, the aqueous phase is irradiated for 15, 30, 45, and 60 min, measuring the absorbance of DB in hexane at each interval. After calculating of the molar extinction coefficient (ε = 10.93 mM·cm^−1^ at 237 nm), the maximum absorbance obtained in the characteristic region of DB in an organic medium in each assay was converted into mass by using the Lambert–Beer law. The mass released by diffusion was subtracted from the total loaded mass to obtain the mass susceptible to being released by laser irradiation.

## 3. Results and Discussion

### 3.1. Dacarbazine Loading into β-Cyclodextrin Forming the New β-Cyclodextrin@dacarbazine Complex

**Figure 2:**
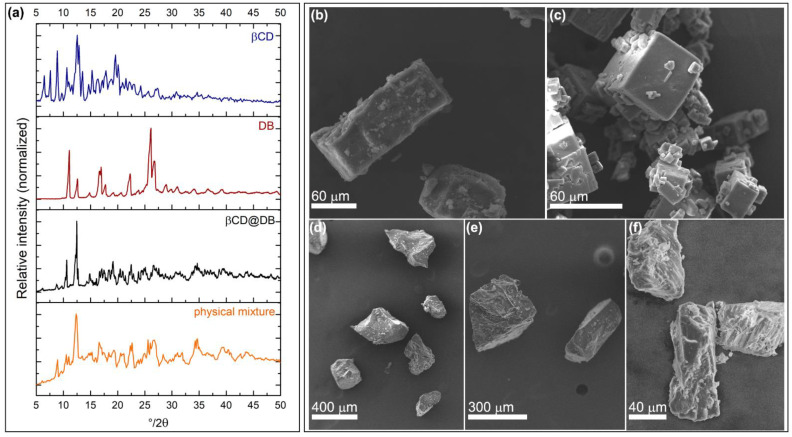
Powder X-ray diffractogram of crystalline powder of βCD, DB, βCD@DB, and βCD-DB physical mixture (**a**). SEM micrograph of βCD (**b**), DB (**c**), βCD@DB (**d**,**e**), and FE-SEM micrography of βCD@DB (**f**).

The drug loading in native βCD was performed using the saturated solutions method, obtaining a precipitate that corresponds to the βCD@DB complex, which was characterized using PXRD and SEM. Figure 2 shows the diffractograms and micrograph for βCD, DB, βCD@DB, and the diffractogram of the physical mixture between βCD and DB.

Peaks at 4°, 6°, 8°, 9°, 13°, and 20° (2θ) of βCD associated with a P-type monoclinic conformation [66], and peaks at 11°, 17°, 22°, 26°, and 27° (2θ) of DB associated with a monoclinic structure of a P2_1/n_ group [67,68], disappeared when the βCD@DB complex was formed. Instead, in the diffractogram of the βCD@DB system appear new peaks, specifically at 10°, 15°, 19°, 23°, and 25° (2θ), that are characteristics of aromatic rings included in βCD matrices [69,70]. The permanence of other peaks corresponding to βCD, in the diffractogram of βCD@DB, reveal the influence of this matrix on the crystalline packing of the complex, while the defined peaks of the DB pattern that disappear in the complex reveal the effective inclusion of the drug. Therefore, crystalline packing arrangement of the complex was different compared to the pure species and represents a new crystalline phase. The physical mixture corresponds to a superposition between the traces of the pure species, confirming that this procedure does not generate interactions that disturb the crystal lattice of any of them (more details in Figure S1, in Supplementary Materials, Section S1).

Different morphologies of βCD, DB, and βCD@DB were observed in SEM micrographs, which reveal that the inclusion phenomenon generates a new crystalline arrangement of the βCD units when the drug is housed in its cavity. Considering that SEM imaging requires the metallization of organic compounds, the complex was covered by gold for 20 s, being a substrate with a higher affinity for this metal than the pure species, which require 100 s of metal deposition. Added to the above, the crystal length analyses show that the βCD@DB complex formation method allows obtaining solid structures that are 13 and 10 times larger compared to the sizes of pure βCD and DB, respectively (additional SEM micrographs, together with size analyses, are detailed in Figure S2 and Table S1 in the Supplementary Materials, Section S1).

Subsequently, a study about the host-guest interactions and stoichiometric ratio of the βCD@DB complex in solution was performed using proton nuclear magnetic resonance (NMR) spectroscopy. Table 1 shows the chemical shifts of βCD and DB protons resulting from the inclusion process. ^1^H-NMR spectra of all species, along with proton assignment, are shown in Figure S3 in the Section S2 of the Supplementary Material.

Protons that are oriented towards the interior of the βCD cavity (H3, H5, and H6) and those close to its openings (OH2, OH3, and OH6) can present chemical shifts due to the inclusion phenomenon [21,71]. Shifts towards lower fields of the H3, H5, and H6 proton signals were observed due to interactions with the highly electronegative atoms of DB. In turn, shifts towards higher fields of the proton signals present in all the hydroxyl groups were observed, probably due to the shielding effect produced by the inclusion of the drug. On the other hand, the shift of the DB proton signals towards higher fields is due to an increase in nearby electron density. This confirms the effective inclusion of the drug in βCD [17,27,28,29]. The stoichiometry of the complex was obtained by comparing the integrals for H1 of βCD regarding CH_3_′, CH_3_′′, CH, and NH of DB, obtaining a stoichiometric ratio of 1:1 (see the details in Figure S4 and Table S2 in the Supplementary Materials, Section S3).

**Figure 3:**
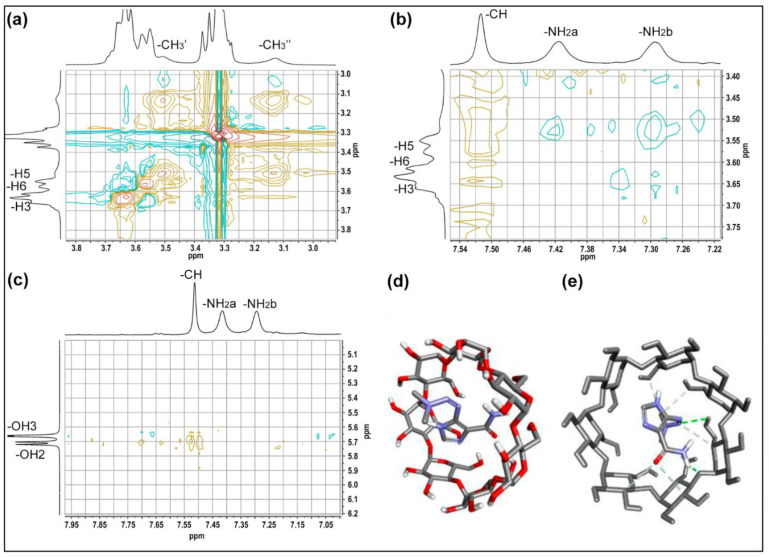
Amplification of the ROESY βCD@DB spectrum showing the interactions of the internal protons of βCD with: (**a**) protons from the -CH_3_ groups of DB; (**b**) proton from the -CH group of the imidazole ring of DB; docking molecular of (**c**) favorable structure of inclusion; and (**d,e**) interactions of the protons of DB with the inner protons of βCD.

Rotational Overhauser Enhancement Spectroscopy (ROESY) complemented with theoretical molecular docking were used to study the interactions and geometry of DB loading [72]. Zoom sections of the two-dimensional spectrum of the βCD@DB complex (a and b) and the most stable conformation obtained by docking (c and d) are shown in Figure 3 (full spectrum in Figure S5 in the Section S4, Supplementary Materials).

Figure 3a–c reflects the interactions between the protons of DB with the internal protons (H3, H5, and H6) and with the protons of the secondary hydroxyls (OH2 and OH3) of βCD. Crossed peaks between CH_3_′ and CH_3_′′ with H5 and H6, together with crossed peaks between NH_2_ and CH with H5 mainly, corroborate the effective inclusion of the drug. The orientation of the guest molecule could be elucidated by the interactions observed between CH and the secondary hydroxyl groups, which would indicate that the region of the imidazole ring is included and oriented towards the wider opening; therefore, the region of the primary amide and methyl groups are oriented toward the narrower opening of βCD. In this sense, molecular modeling is frequently used to support the structural elucidation of inclusion complexes [61,73]. Figure 3d,e corresponds to a theoretical arrangement of DB in the matrix; here the complete inclusion of the drug was observed, with specific interactions between the protons of the CH_3_ groups with H5 and H6, and the protons of the CH groups with H3, H5, and secondary hydroxyl groups, confirming a dynamic inclusion of DB, which is oriented towards the narrower opening of βCD.

Finally, to determine the pharmaceutical parameters of relevance, DS, CE, and *Ka*, the phase solubility method was used [57]. The solubility graph indicates that the formation kinetics correspond to an AL type diagram, confirming a 1:1 stoichiometry inclusion compound, which represents a βCD loading capacity of 16% for DB (see the details in Figures S6 and S7 in the Supplementary Materials, Section S5). An increase in the aqueous solubility of the drug was observed with increasing concentrations of βCD. The DS value for the complex was 0.023; the CE was 0.024; and *Ka* was 80 M^−1^. These data constitute the basis for evaluating the feasibility of a future pharmaceutical formulation. In general, values greater than 2000 M^−1^ indicate a limitation in the pharmaceutical formulation, such as poor pharmacokinetics, since the drug release rates can be affected. Meanwhile, *Ka* with lower values of 50 M^−1^ also present limitations since they have low stability and do not release the drug at its site of action [74]. In this sense, to ensure the permanence of the drug in the transport system, the use of AuNPs becomes relevant. It has previously been reported that the presence of AuNPs interacting with the βCD-drug complex can decrease the degree of dissociation of the drug in solution, controlling its release by means of a stimulus such as laser irradiation [29]. 

It is known that AuNPs can absorb the energy emitted by an external source, such as a laser, which is subsequently released in the form of heat together with the molecules that are close to its surface as another consequence of this photothermal effect [34,75,76]. Notably, this has been demonstrated for a drug included in AuNPs- and βCD-based systems using laser irradiation [29,31]. Therefore, in addition to generating localized hyperthermia, AuNPs are therapeutic agents, especially in cancer, where they also act as nanocarriers for the antitumor drug, which can be released if localized energy is irradiated.

### 3.2. Functionalization of Gold Nanoparticle Surface with β-Cyclodextrin@dacarbazine Complex

**Figure 4:**
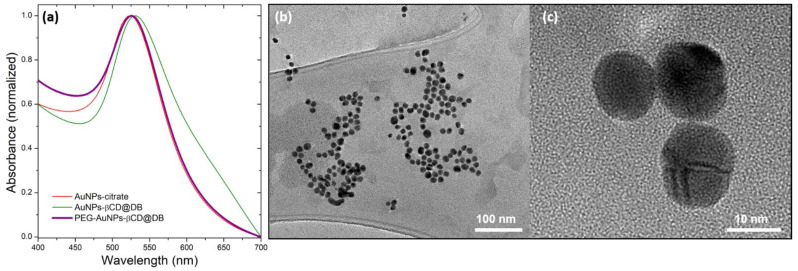
(**a**) UV-vis spectra of AuNPs stabilized with citrate: βCD@DB and PEG-βCD@DB. HR-TEM of the PEG-AuNPs-βCD@DB system: (**b**) wide view and (**c**) zoom-in.

To form the nanocarrier based on AuNPs, they were synthesized following the Turkevich method [62], and then, the citrate ligand was exchanged for the βCD@DB complex. To increase the colloidal stability of the system, CH_3_O-PEG_(5000)_-SH molecules were incorporated, forming the new PEG-AuNPs-βCD@DB nanosystem. The AuNPs obtained and functionalized in their different stages were characterized by UV-vis spectroscopy, dynamic light scattering (DLS), Zeta potential, and transmission electron microscopy (TEM). Figure 4 shows the UV-VIS spectra of AuNPs-citrate, AuNPs-βCD@DB, and PEG-AuNPs-βCD@DB, and HR-TEM micrographs of AuNPs stabilized with PEG and βCD@DB system. In addition, Table 2 shows the results obtained from the different techniques used in these nanosystems.

A bathochromic displacement at 525 nm of the plasmon of the AuNPs was observed when citrate was replaced by the PEG-complex as stabilizer due to the new interaction of the electrons of the nanoparticles with functional groups SH (from PEG) and NH_2_ (from DB in βCD), as has been reported [28,45,77]. In addition, an increase in hydrodynamic diameter from 45 to 65 nm and PDI from 0.3 to 0.4 for the PEG-AuNPs-βCD@DB system comparing to AuNPs-citrate was observed using DLS. This increase in the solvation sphere was attributed to the replacement of citrate by PEG and βCD@DB, which have greater size and mass. In this sense, the change in surface charge from −43 mV to −29 mV corroborates the effective exchange of the stabilizer. HR-TEM images of the PEG-AuNPs-βCD@DB nanosystem show the spherical shape of the nanoparticles with an average diameter of 12 nm, which is the same as for the as-synthesized AuNPs using citrate. These results confirm that the plasmonic and superficial properties’ changes can be attributed to the replacement of the stabilizer without a process of aggregation or interparticle coupling occurring (see the details in Supplementary Materials, Section S6). After the PEG-AuNPs-βCD@DB purification process, the drug content of the extracted supernatant was analyzed to evaluate the loading efficiency of the AuNPs. According to this, 99% of the initial mass of DB remains in the βCD@DB complex loaded to the nanosystem. This reaffirms that the drug, previously included in βCD, is interacting with the surface gold atoms of the nanoparticles (see Table S3 in Supplementary Materials, Section S6). 

**Figure 5:**
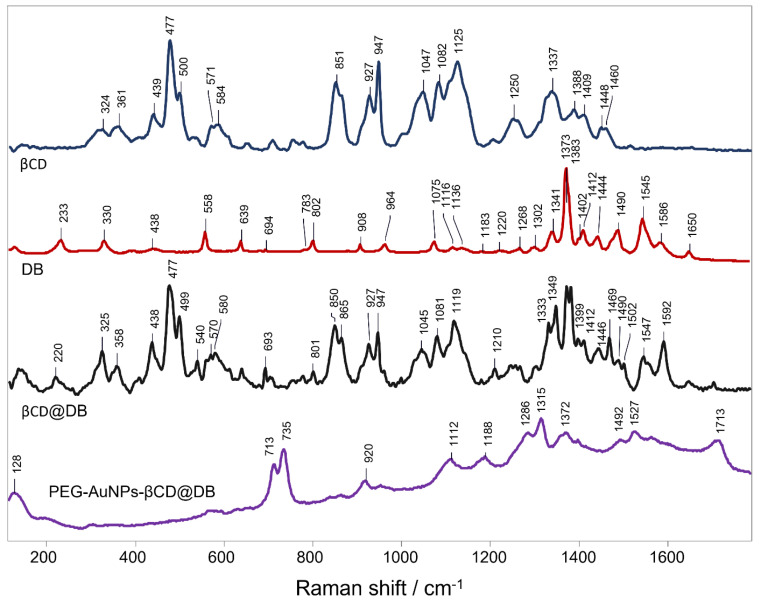
Raman spectra of βCD, DB, and βCD@DB, and SERS spectra of PEG-AuNPs-βCD@DB.

The inclusion phenomenon of DB in βCD and its interaction with AuNPs generate changes in the vibrational modes of the constituting molecules, which are associated with modifications in the intensity or spectral position of their signals in the Raman spectrum. Next, the Raman and SERS techniques were used to study the disposition and interaction of the drug loaded in the nanosystem. Figure 5 shows the Raman spectra of DB, βCD, βCD@DB, and PEG-AuNPs-βCD@DB. The DB vibrational mode assignments in the Raman spectra are attached in Table S4 in Section S7, Supplementary Materials. 

In the DB spectrum, signals at 325, 540, 1210, 1547, and 1592 cm^−1^ associated with vibrational modes of azide and amide can be observed. These signals were also observed in βCD@DB spectra. The signal at 1372 cm^−1^ in the DB spectra, associated with the imidazole bending and azide stretching, shows a splitting in βCD@DB spectra, suggesting that the chemical environment was different for both sections, probably because the imidazole was found interacting with the βCD interior while the azide was arranged outward. The loss of the remaining DB signals in the βCD@DB spectrum was due to the vibrational restraint caused by the inclusion phenomenon. This effect was also supported by the IR spectroscopy analysis (see the details in Figure S12 in Section S7, Supplementary Materials). 

On the other hand, in the SERS spectrum of PEG-AuNPs-βCD@DB, amplified signals of DB were observed at 713 and 754 cm^−1^, associated with N-C-O scissoring and C-N azide in-plane bending, as well as signals at 1286 and 1314 cm^−1^, associated with imidazole in-plane vibrations. In particular, the C=O stretching signal at 1712 cm^−1^ appears in PEG-AuNPs-βCD@DB but not in the DB spectrum. The decrease of signals at 1372, 1492, and 1527 cm^−1^, associated mainly with imidazole, azide, and N-(CH_3_)_2_ out-of-plane vibrations regarding DB spectra, was observed in conjunction with the loss of signals at 1210 and 1592 cm^−1^, associated principally with NH_2_. These changes suggest that the dynamic inclusion of DB in βCD, discussed in ROESY and docking, allows its mobility and orientation to suit its interaction with the gold surface. A perpendicular arrangement regarding the molecular plane of DB on the AuNPs’ surface through its interaction via the NH_2_ group was proposed [17,78]. Notably, the signal in the 130 cm^−1^ sector suggests the formation of the thiol-gold bond of the PEG used as a stabilizer [79,80].

### 3.3. Dacarbazine Controlled Release Using Laser Irradiation

**Figure 6:**
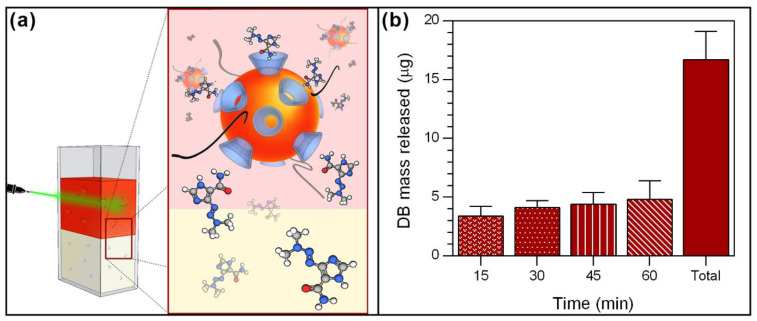
(**a**) A schematic representation of the setup used to irradiate the nanosystem using a laser at 532 nm. (**b**) Average mass of DB released at different time intervals and in total using laser irradiation on the PEG-AuNPs-βCD@DB nanosystem.

The photothermal effect of AuNPs has been widely studied for controlled drug release and to produce hyperthermia, which effectively and locally kills cancer cells [29,31,32,47,81,82]. This effect can be generated by the application of energy from an external light source with a suitable wavelength and intensity, such as a laser. As a proof of concept, the controlled release of DB triggered by laser irradiation onto AuNPs present in the nanosystem was tested. Figure 6 shows the amount of DB mass released at different time intervals and the total using a continuous laser at 532 nm.

A two-phase system consisting of an aqueous phase with PEG-AuNPs-βCD@DB and an organic phase was developed. In the latter, the drug is expected to diffuse almost freely after its release from the nanosystem due to its mainly non-polar character. First, a passive diffusion of 2 μg was observed. This was subtracted from the total to highlight only the stimulated release. Then, laser irradiation triggers the release of DB from the nanosystem, reaching 3.4 μg after 15 min. A tendency to increase the amount of DB released as a function of time was established, achieving a maximum release of 4.8 μg from 45 to 60 min of laser irradiation. Subsequently, a total release of 16.7 ± 3.9 μg was obtained after 60 min of irradiation, which corresponds to 42.9 ± 4.1% of the total DB loaded in the nanosystem; this represents a concentration of 55.7 μg/mL (see the details in Table S5 in Section S8, Supplementary Materials). The release profile and the times involved were equivalent to previous reports on molecules released from systems based on cyclodextrins and AuNPs [29,31]. Considering the cytotoxicity of DB expressed in terms of IC50 (the drug concentration required for killing 50% of the B16F1 mouse melanoma cancer cell line), the value that has been reported is 0.48 mg/mL [83], hence the amount released from the nanosystem may be adequate. Accordingly, the laser-triggered controlled release of DB from the PEG-AuNPs-βCD@DB nanosystem could reach optimized concentrations at the site of action compared to free DB. Thus, it is proposed that the PEG-AuNPs-βCD@DB system may be a promising candidate for use in cancer therapy.

## 4. Conclusions

The new inclusion complex between DB and βCD in a solid-state was successfully obtained. DB presents a dynamic inclusion into βCD, remaining stable in solution in a 1:1 ratio. Notably, βCD shows a loading capacity of 16% with a suitable association constant and an increase in its aqueous solubility. These pharmaceutical parameters facilitate its projection and comparison with other possible complexations of DB.

In turn, citrate-coated AuNPs with an average diameter of 12 nm were obtained, which are suitable for applications in biological systems. Subsequently, the citrate molecules were effectively exchanged by the βCD@DB complexes and PEG, resulting in a new nanosystem that was stable over time and had a high loading efficiency.

Laser irradiation assays showed that the PEG-AuNPs-βCD@DB nanosystem is able to release drug in a controlled manner through the excitation caused by an incident electromagnetic wave, confirming the generation of the surface plasmon resonance effect and having a total average release amount corresponding to 43% with respect to the mass of DB contained in the nanosystem. According to the above, it is understood that the DB loading in this new nanocarrier based on βCD and AuNPs has the potential to be tested in biological systems as an alternative improvement to current cancer treatments. Studies of DB loaded to the new nanosystem, such as evaluation of cytotoxicity, membrane permeability, and therapeutic potential in in vitro and in vivo cancer models, are considered necessary to continue with the validation of this strategy for drug delivery applications.

## Figures and Tables

**Table 1:** Proton assignments and ^1^H-NMR chemical shifts of βCD, DB, and βCD@DB.

Proton	δβCD (ppm)	δβCD@DB (ppm)	Δδ (ppm)	Proton	δDB (ppm)	δβCD@DB (ppm)	Δδ (ppm)
-H3	3.648	3.657	0.009	-NH	12.617	12.591	−0.026
-H5	3.555	3.561	0.006	-CH_3′_	3.518	3.504	−0.014
-H6	3.617	3.623	0.006	-CH_3′_’	3.131	3.126	−0.005
-OH2	5.735	5.711	−0.024	-CH	7.527	7.520	−0.007
-OH3	5.680	5.662	−0.018	-NH_2_a,b	7.451	7.416	−0.035
-OH6	4.479	4.436	−0.043		7.307	7.294	−0.013

**Table 2:** Wavelengths of the maximum absorbance (λ_max_), hydrodynamic diameter (D_H_), polydispersity index (PDI), surface charge, and diameter according to TEM for AuNPs with different stabilizers.

Nanosystem	λ_max_ (nm)	D_H_ (nm)	PDI	Surface Charge (mV)	TEM Diameter (nm)
AuNPs-citrate	523	45 ± 11	0.33	−43 ± 4	12 ± 4
PEG-AuNPs-βCD@DB	525	65 ± 25	0.41	−29 ± 4	12 ± 3

## Data Availability

Not applicable.

## Supplementary Materials

The following supporting information can be downloaded at: https://www.mdpi.com/article/10.3390/pharmaceutics15020458/s1, Figure S1: Powder X-ray diffractogram zoom of the β-cyclodextrin@dacarbazine complex (black) and physical mixture between β-cyclodextrin and dacarbazine (orange). Figure S2: SEM micrograph of β-cyclodextrin (a), dacarbazine (b), and field emission SEM micrography of β-cyclodextrin@dacarbazine (c). Figure S3: (a) Molecular structure of dacarbazine in its two tautomeric forms; (b) Cone-type model of β-cyclodextrin indicating the orientation of its protons, which are represented for a unit of glucose. ^1^H-NMR spectra of: (c) β-cyclodextrin; (d) dacarbazine; and (e) β-cyclodextrin@dacarbazine complex, obtained in DMSO-d_6_. Figure S4: Integrated signals of dacarbazine and β-cyclodextrin protons in the ^1^H-NMR spectrum of the β-cyclodextrin@dacarbazine complex. Figure S5: ROESY spectra of the β-cyclodextrin@dacarbazine complex. Figure S6: Graph of maximum absorbance at 237 nm versus dacarbazine concentration. Figure S7: Graph of concentrations of: solubilized dacarbazine versu ꞵ-cyclodextrin used. Figure S8: Hydrodynamic diameter distribution by intensity of gold nanoparticles stabilized with: citrate (red) and PEG-β-cyclodextrin@dacarbazine (purple). Figure S9: TEM of gold nanoparticles stabilized with citrate. Figure S10: Size histogram of gold nanoparticles stabilized with citrate. Figure S11: Size histogram of gold nanoparticles stabilized with β-cyclodextrin@dacarbazine and PEG. Figure S12: FT-IR spectra of β-cyclodextrin, dacarbazine, and the β-cyclodextrin@dacarbazine complex. Table S1: Average length values of the complex and its pure species, obtained from the SEM images. Table S2: Values of the integrated signals of dacarbazine and β-cyclodextrin using the ^1^H-NMR spectrum of the complex formed. Table S3: Mass of dacarbazine in the supernatant, amount of dacarbazine loaded on gold nanoparticles, and efficiency of dacarbazine included in β-cyclodextrin and loaded on gold nanoparticles. Table S4: Theorical Raman shift, Raman shift observed in the dacarbazine spectrum and the corresponding vibrational mode. Table S5: Mass released of dacarbazine at each time interval, sum of all mass released, and percentage of average cumulant released mass.

## References

[1] Sung H., Ferlay J., Siegel R.L., Laversanne M., Soerjomataram I., Jemal A., Bray F. (2021). Global Cancer Statistics 2020: GLOBOCAN Estimates of Incidence and Mortality Worldwide for 36 Cancers in 185 Countries. CA. Cancer J. Clin..

[2] Cheng Z., Li M., Dey R., Chen Y. (2021). Nanomaterials for cancer therapy: Current progress and perspectives. J. Hematol. Oncol..

[3] Cho K., Wang X., Nie S., Chen Z., Shin D.M. (2008). Therapeutic nanoparticles for drug delivery in cancer. Clin. Cancer Res..

[4] Serrone L., Zeuli M., Sega F.M., Cognetti F. (2000). Dacarbazine-based chemotherapy for metastatic melanoma: Thirty-year experience overview. J. Exp. Clin. Cancer Res..

[5] Eggermont A.M.M., Kirkwood J.M. (2004). Re-evaluating the role of dacarbazine in metastatic melanoma: What have we learned in 30 years?. Eur. J. Cancer.

[6] Niemeijer N.D., Alblas G., Van Hulsteijn L.T., Dekkers O.M., Corssmit E.P.M. (2014). Chemotherapy with cyclophosphamide, vincristine and dacarbazine for malignant paraganglioma and pheochromocytoma: Systematic review and meta-analysis. Clin. Endocrinol..

[7] Chapman P.B., Einhorn L.H., Meyers M.L., Saxman S., Destro A.N., Panageas K.S., Begg C.B., Agarwala S.S., Schuchter L.M., Ernstoff M.S. (1999). Phase III multicenter randomized trial of the Dartmouth regimen versus dacarbazine in patients with metastatic melanoma. J. Clin. Oncol..

[8] Lev D.C., Onn A., Melinkova V.O., Miller C., Stone V., Ruiz M., McGary E.C., Ananthaswamy H.N., Price J.E., Bar-Eli M. (2004). Exposure of melanoma cells to dacarbazine results in enhanced tumor growth and metastasis in vivo. J. Clin. Oncol..

[9] Al-Badr A.A., Alodhaib M.M. (2016). Dacarbazine. Profiles of Drug Substances, Excipients and Related Methodology.

[10] Pourahmad J., Amirmostofian M., Kobarfard F., Shahraki J. (2009). Biological reactive intermediates that mediate dacarbazine cytotoxicity. Cancer Chemother. Pharmacol..

[11] Asahi M., Matsushita R., Kawahara M., Ishida T., Emoto C., Suzuki N., Kataoka O., Mukai C., Hanaoka M., Ishizaki J. (2010). Causative agent of vascular pain among photodegradation products of dacarbazine. J. Pharm. Pharmacol..

[12] Besse J.P., Latour J.F., Garric J. (2012). Anticancer drugs in surface waters. What can we say about the occurrence and environmental significance of cytotoxic, cytostatic and endocrine therapy drugs?. Environ. Int..

[13] Booker V., Halsall C., Llewellyn N., Johnson A., Williams R. (2014). Prioritising anticancer drugs for environmental monitoring and risk assessment purposes. Sci. Total Environ..

[14] Real D.A., Bolaños K., Priotti J., Yutronic N., Kogan M.J., Sierpe R., Donoso-González O. (2021). Cyclodextrin-modified nanomaterials for drug delivery: Classification and advances in controlled release and bioavailability. Pharmaceutics.

[15] Gadade D.D., Pekamwar S.S. (2020). Cyclodextrin based nanoparticles for drug delivery and theranostics. Adv. Pharm. Bull..

[16] Park C., Youn H., Kim H., Noh T., Kook Y.H., Oh E.T., Park H.J., Kim C. (2009). Cyclodextrin-covered gold nanoparticles for targeted delivery of an anti-cancer drug. J. Mater. Chem..

[17] Donoso-González O., Lodeiro L., Aliaga Á.E., Laguna-Bercero M.A., Bollo S., Kogan M.J., Yutronic N., Sierpe R. (2021). Functionalization of gold nanostars with cationic β-cyclodextrin-based polymer for drug co-loading and sers monitoring. Pharmaceutics.

[18] Crini G., Fourmentin S., Fenyvesi É., Torri G., Fourmentin M., Morin-Crini N. (2018). Cyclodextrins, from molecules to applications. Environ. Chem. Lett..

[19] Kurkov S.V., Loftsson T. (2013). Cyclodextrins. Int. J. Pharm..

[20] Davis M.E., Brewster M.E. (2004). Cyclodextrin-based pharmaceutics: Past, present and future. Nat. Rev. Drug Discov..

[21] Szejtli J. (1998). Introduction and General Overview of Cyclodextrin Chemistry. Chem. Rev..

[22] Wankar J., Kotla N.G., Gera S., Rasala S., Pandit A., Rochev Y.A. (2020). Recent Advances in Host–Guest Self-Assembled Cyclodextrin Carriers: Implications for Responsive Drug Delivery and Biomedical Engineering. Adv. Funct. Mater..

[23] Zhang D., Lv P., Zhou C., Zhao Y., Liao X., Yang B. (2019). Cyclodextrin-based delivery systems for cancer treatment. Mater. Sci. Eng. C.

[24] Tian B., Hua S., Liu J. (2020). Cyclodextrin-based delivery systems for chemotherapeutic anticancer drugs: A review. Carbohydr. Polym..

[25] Loftsson T., Másson M., Brewster M.E. (2004). Self-Association of Cyclodextrins and Cyclodextrin Complexes. J. Pharm. Sci..

[26] Saokham P., Muankaew C., Jansook P., Loftsson T. (2018). Solubility of cyclodextrins and drug/cyclodextrin complexes. Molecules.

[27] Sierpe R., Noyong M., Simon U., Aguayo D., Huerta J., Kogan M.J., Yutronic N. (2017). Construction of 6-thioguanine and 6-mercaptopurine carriers based on βcyclodextrins and gold nanoparticles. Carbohydr. Polym..

[28] Asela I., Noyong M., Simon U., Andrades-Lagos J., Campanini-Salinas J., Vásquez-Velásquez D., Kogan M., Yutronic N., Sierpe R. (2017). Gold nanoparticles stabilized with βcyclodextrin-2-amino-4-(4-chlorophenyl) thiazole complex: A novel system for drug transport. PLoS One.

[29] Sierpe R., Lang E., Jara P., Guerrero A.R., Chornik B., Kogan M.J., Yutronic N. (2015). Gold Nanoparticles Interacting with β-Cyclodextrin-Phenylethylamine Inclusion Complex: A Ternary System for Photothermal Drug Release. ACS Appl. Mater. Interfaces.

[30] Asela I., Donoso-González O., Yutronic N., Sierpe R. (2021). β-cyclodextrin-based nanosponges functionalized with drugs and gold nanoparticles. Pharmaceutics.

[31] Silva N., Riveros A., Yutronic N., Lang E., Chornik B., Guerrero S., Samitier J., Jara P., Kogan M. (2018). Photothermally Controlled Methotrexate Release System Using β-Cyclodextrin and Gold Nanoparticles. Nanomaterials.

[32] Riley R.S., Day E.S. (2017). Gold nanoparticle-mediated photothermal therapy: Applications and opportunities for multimodal cancer treatment. Wiley Interdiscip. Rev. Nanomed. Nanobiotechnol..

[33] Her S., Jaffray D.A., Allen C. (2017). Gold nanoparticles for applications in cancer radiotherapy: Mechanisms and recent advancements. Adv. Drug Deliv. Rev..

[34] Kim H.S., Lee D.Y. (2017). Photothermal therapy with gold nanoparticles as an anticancer medication. J. Pharm. Investig..

[35] Wuithschick M., Birnbaum A., Witte S., Sztucki M., Vainio U., Pinna N., Rademann K., Emmerling F., Kraehnert R., Polte J. (2015). Turkevich in New Robes: Key Questions Answered for the Most Common Gold Nanoparticle Synthesis. ACS Nano.

[36] Chhour P., Naha P.C., Cheheltani R., Benardo B., Mian S., Cormode D.P. (2016). Gold nanoparticles for biomedical applications: Synthesis and in vitro evaluation. Methods in Pharmacology and Toxicology.

[37] Zhao P., Li N., Astruc D. (2013). State of the art in gold nanoparticle synthesis. Coord. Chem. Rev..

[38] Pan Y., Neuss S., Leifert A., Fischler M., Wen F., Simon U., Schmid G., Brandau W., Jahnen-Dechent W. (2007). Size-dependent cytotoxicity of gold nanoparticles. Small.

[39] Murphy C.J., Gole A.M., Stone J.W., Sisco P.N., Alkilany A.M., Goldsmith E.C., Baxter S.C. (2008). Gold nanoparticles in biology: Beyond toxicity to cellular imaging. Acc. Chem. Res..

[40] Cheng Y., Samia A.C., Meyers J.D., Panagopoulos I., Fei B., Burda C. (2008). Highly efficient drug delivery with gold nanoparticle vectors for in vivo photodynamic therapy of cancer. J. Am. Chem. Soc..

[41] Duncan B., Kim C., Rotello V.M. (2010). Gold nanoparticle platforms as drug and biomacromolecule delivery systems. J. Control. Release.

[42] Guerrero S., Araya E., Fiedler J.L., Arias J.I., Adura C., Albericio F., Giralt E., Arias J.L., Fernndez M.S., Kogan M.J. (2010). Improving the brain delivery of gold nanoparticles by conjugation with an amphipathic peptide. Nanomedicine.

[43] Li S.D., Huang L. (2008). Pharmacokinetics and biodistribution of nanoparticles. Proceedings of the Molecular Pharmaceutics.

[44] Setyawati M.I., Tay C.Y., Bay B.H., Leong D.T. (2017). Gold Nanoparticles Induced Endothelial Leakiness Depends on Particle Size and Endothelial Cell Origin. ACS Nano.

[45] Khlestkin V.K., Polienko J.F., Voinov M.A., Smirnov A.I., Chechik V. (2008). Interfacial surface properties of thiol-protected gold nanoparticles: A molecular probe EPR approach. Langmuir.

[46] Ajnai G., Chiu A., Kan T., Cheng C.C., Tsai T.H., Chang J. (2014). Trends of Gold Nanoparticle-based Drug Delivery System in Cancer Therapy. J. Exp. Clin. Med..

[47] Guerrero A.R., Hassan N., Escobar C.A., Albericio F., Kogan M.J., Araya E. (2014). Gold nanoparticles for photothermally controlled drug release. Nanomedicine.

[48] Nicol J.R., Dixon D., Coulter J.A. (2015). Gold nanoparticle surface functionalization: A necessary requirement in the development of novel nanotherapeutics. Nanomedicine.

[49] Han G., Ghosh P., De M., Rotello V.M. (2007). Drug and gene delivery using gold nanoparticles. Nanobiotechnology.

[50] Ghosh P., Han G., De M., Kim C.K., Rotello V.M. (2008). Gold nanoparticles in delivery applications. Adv. Drug Deliv. Rev..

[51] Balfourier A., Kolosnjaj-Tabi J., Luciani N., Carn F., Gazeau F., Murphy C.J. (2020). Gold-based therapy: From past to present. Proc. Natl. Acad. Sci. USA.

[52] Bergen J.M., Von Recum H.A., Goodman T.T., Massey A.P., Pun S.H. (2006). Gold nanoparticles as a versatile platform for optimizing physicochemical parameters for targeted drug delivery. Macromol. Biosci..

[53] Chandran P.R., Thomas R.T. (2015). Gold Nanoparticles in Cancer Drug Delivery. Nanotechnology Applications for Tissue Engineering.

[54] Jia L., Zhang P., Sun H., Dai Y., Liang S., Bai X., Feng L. (2021). Optimization of nanoparticles for smart drug delivery: A review. Nanomaterials.

[55] Coleman A.W., Nicolis I., Keller N., Dalbiez J.P. (1992). Aggregation of cyclodextrins: An explanation of the abnormal solubility of β-cyclodextrin. J. Incl. Phenom. Mol. Recognit. Chem..

[56] Omar S.M., Ibrahim F., Ismail A. (2020). Formulation and evaluation of cyclodextrin-based nanosponges of griseofulvin as pediatric oral liquid dosage form for enhancing bioavailability and masking bitter taste. Saudi Pharm. J..

[57] Higuchi T., Connors K.A., Reilly C.N. (1965). Phase Solubility Techniques. Advances in Analytical Chemistry and Instrumentation.

[58] Trott O., Olson A.J. (2009). AutoDock Vina: Improving the speed and accuracy of docking with a new scoring function, efficient optimization, and multithreading. J. Comput. Chem..

[59] Giastas P., Yannakopoulou K., Mavridis I.M. (2003). Molecular structures of the inclusion complexes β-cyclodextrin-1,2-bis(4-aminophenyl)ethane and β-cyclodextrin-4,4′-diaminobiphenyl; packing of dimeric β-cyclodextrin inclusion complexes. Acta Crystallogr. Sect. B Struct. Sci..

[60] Robinson-Duggon J., McTiernan C.D., Muñoz M., Guerra D., Escobar Álvarez E., Andrade-Villalobos F., Fierro A., Edwards A.M., Alarcon E.I., Fuentealba D. (2021). Biosupramolecular complexes of amphiphilic photosensitizers with human serum albumin and cucurbit[7]uril as carriers for photodynamic therapy. J. Photochem. Photobiol. B Biol..

[61] Li T., Guo R., Zong Q., Ling G. (2022). Application of molecular docking in elaborating molecular mechanisms and interactions of supramolecular cyclodextrin. Carbohydr. Polym..

[62] Turkevich J., Stevenson P.C., Hillier J. (1951). A study of the nucleation and growth processes in the synthesis of colloidal gold. Discuss. Faraday Soc..

[63] Liu X., Atwater M., Wang J., Huo Q. (2007). Extinction coefficient of gold nanoparticles with different sizes and different capping ligands. Colloids Surf. B Biointerfaces.

[64] Near R.D., Hayden S.C., Hunter R.E., Thackston D., El-Sayed M.A. (2013). Rapid and efficient prediction of optical extinction coefficients for gold nanospheres and gold nanorods. J. Phys. Chem. C.

[65] Frisch M.J., Trucks G.W., Schlegel H.B., Scuseria G.E., Robb M.A., Cheeseman J.R., Scalmani G., Barone V., Mennucci B., Petersson H. (2013). Gaussian 09, Revision D.01.

[66] Aree T., Chaichit N. (2003). A new crystal form of β-cyclodextrin-ethanol inclusion complex: Channel-type structure without long guest molecules. Carbohydr. Res..

[67] Bei D., Zhang T., Murowchick J.B., Youan B.B.C. (2010). Formulation of dacarbazine-loaded cubosomes. Part III. physicochemical characterization. AAPS PharmSciTech.

[68] Freeman H.C., Hutchinson N.D. (1979). The crystal structure of the anti-tumor agent 5-(3,3-dimethyl-1-triazenyl)imidazole-4-carboxamide (NSC-45388). Acta Crystallogr. Sect. B Struct. Crystallogr. Cryst. Chem..

[69] Sala A., Hoossen Z., Bacchi A., Caira M.R. (2021). Two crystal forms of a hydrated 2:1 b-cyclodextrin× fluconazole complex: Single crystal x-ray structures, dehydration profiles, and conditions for their individual isolation. Molecules.

[70] Dang Z., Xin Song L., Qing Guo X., Yun Du F., Yang J., Yang J. (2011). Applications of Powder X-Ray Diffraction to Inclusion Complexes of Cyclodextrins. Curr. Org. Chem..

[71] Uekama K., Hirayama F., Irie T. (1998). Cyclodextrin drug carrier systems. Chem. Rev..

[72] Rhodes C.J. (2017). Magnetic resonance spectroscopy. Sci. Prog..

[73] Cheriet M., Djemil R., Khellaf A., Khatmi D. (2022). Dopamine Family Complexes With β-Cyclodextrin: Molecular Docking Studies. Polycycl. Aromat. Compd..

[74] Rao V.M., Stella V.J. (2003). When can cyclodextrins be considered for solubilization purposes?. J. Pharm. Sci..

[75] Tao Y., Chan H.F., Shi B., Li M., Leong K.W. (2020). Light: A Magical Tool for Controlled Drug Delivery. Adv. Funct. Mater..

[76] Elahi N., Kamali M., Baghersad M.H. (2018). Recent biomedical applications of gold nanoparticles: A review. Talanta.

[77] Wang W., Wei Q.Q., Wang J., Wang B.C., Zhang S.H., Yuan Z. (2013). Role of thiol-containing polyethylene glycol (thiol-PEG) in the modification process of gold nanoparticles (AuNPs): Stabilizer or coagulant?. J. Colloid Interface Sci..

[78] Aroca R. (2007). Surface-Enhanced Vibrational Spectroscopy.

[79] Chadha R., Das A., Kapoor S., Maiti N. (2021). Surface-induced dimerization of 2-thiazoline-2-thiol on silver and gold nanoparticles: A surface enhanced Raman scattering (SERS) and density functional theoretical (DFT) study. J. Mol. Liq..

[80] Nyamekye C.K.A., Weibel S.C., Smith E.A. (2021). Directional Raman scattering spectra of metal–sulfur bonds at smooth gold and silver substrates. J. Raman Spectrosc..

[81] Hwang S., Nam J., Jung S., Song J., Doh H., Kim S. (2014). Gold nanoparticle-mediated photothermal therapy: Current status and future perspective. Nanomedicine.

[82] Bolaños K., Sánchez-Navarro M., Giralt E., Acosta G., Albericio F., Kogan M.J., Araya E. (2021). NIR and glutathione trigger the surface release of methotrexate linked by Diels-Alder adducts to anisotropic gold nanoparticles. Mater. Sci. Eng. C.

[83] Hafeez A., Kazmi I. (2017). Dacarbazine nanoparticle topical delivery system for the treatment of melanoma. Sci. Rep..

