# Supplementary material for: Optimizing Dacarbazine Therapy: Design of a Laser-Triggered Delivery System Based on β-Cyclodextrin and Plasmonic Gold Nanoparticles
Source: Pharmaceutics. 2023 Jan 30;15(2):458. doi: 10.3390/pharmaceutics15020458 (PMC9960602; doi:10.3390/pharmaceutics15020458)
Supplement: Supplementary file 1 [file pharmaceutics-15-00458-s001.zip › pharmaceutics-2150238-supplementary.pdf]

S1. XRD zoom and additionally SEM micrographs

Figure S1 shows the XRD zoom of β-cyclodextrin@dacarbazine complex and physical mixture of the pure species.

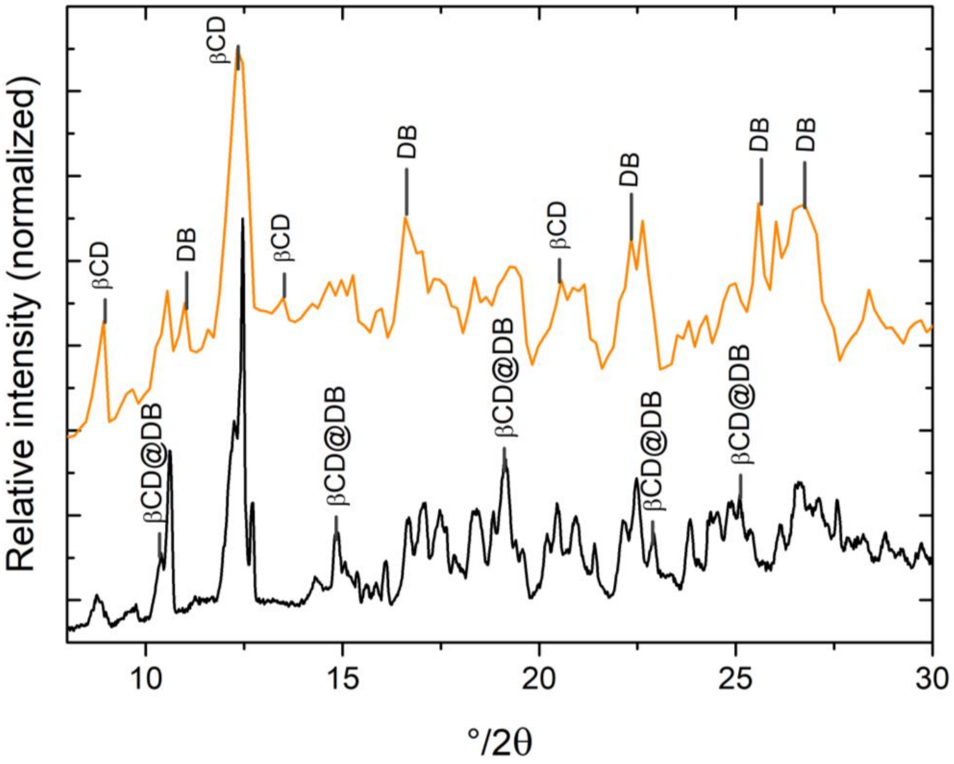

Figure S1. Powder X-ray diffractogram zoom of the β-cyclodextrin@dacarbazine complex (black) and physical mixture between β-cyclodextrin and dacarbazine (orange).

Figure S2 shows the SEM micrograph of β-cyclodextrin and dacarbazine. Table S1 shows the average crystal length values of β-cyclodextrin, dacarbazine, and β-cyclodextrin@dacarbazine, which were calculated using the SEM images of the article and the supplementary material. Furthermore, this length measurement was made only for the largest crystals.

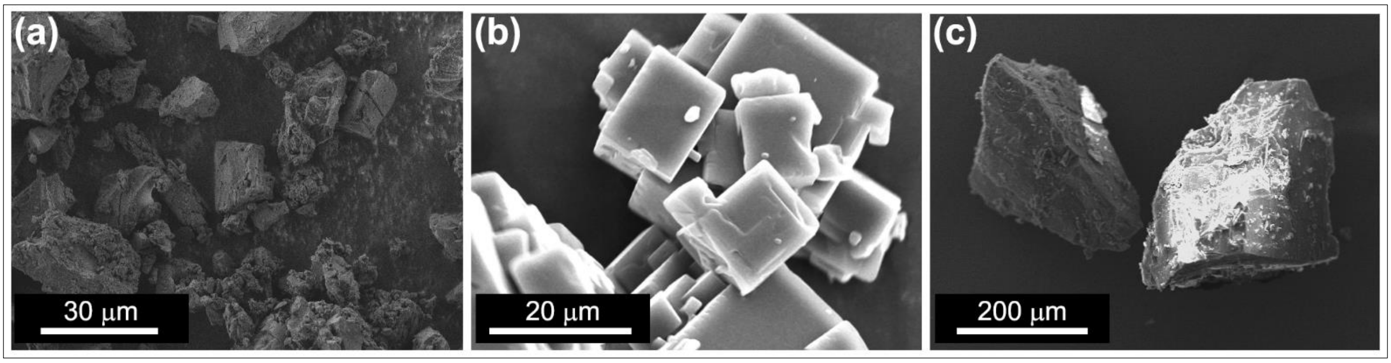

Figure S2. SEM micrograph of β-cyclodextrin (a), dacarbazine (b), and field emission SEM micrography of β-cyclodextrin@dacarbazine (c).

Table S1. Average length values of the complex and its pure species, obtained from the SEM images

Cristalline compound	Average length and SD (mm)
β-cyclodextrin	23 ± 8
Dacarbazine	30 ± 17
β-cyclodextrin@dacarbazine	302 ± 122

S2. ^{1}H-NMR spectra

Figure S3 shows the ^{1}H-NMR spectra of the pure species and the β-cyclodextrin@dacarbazine complex formed.

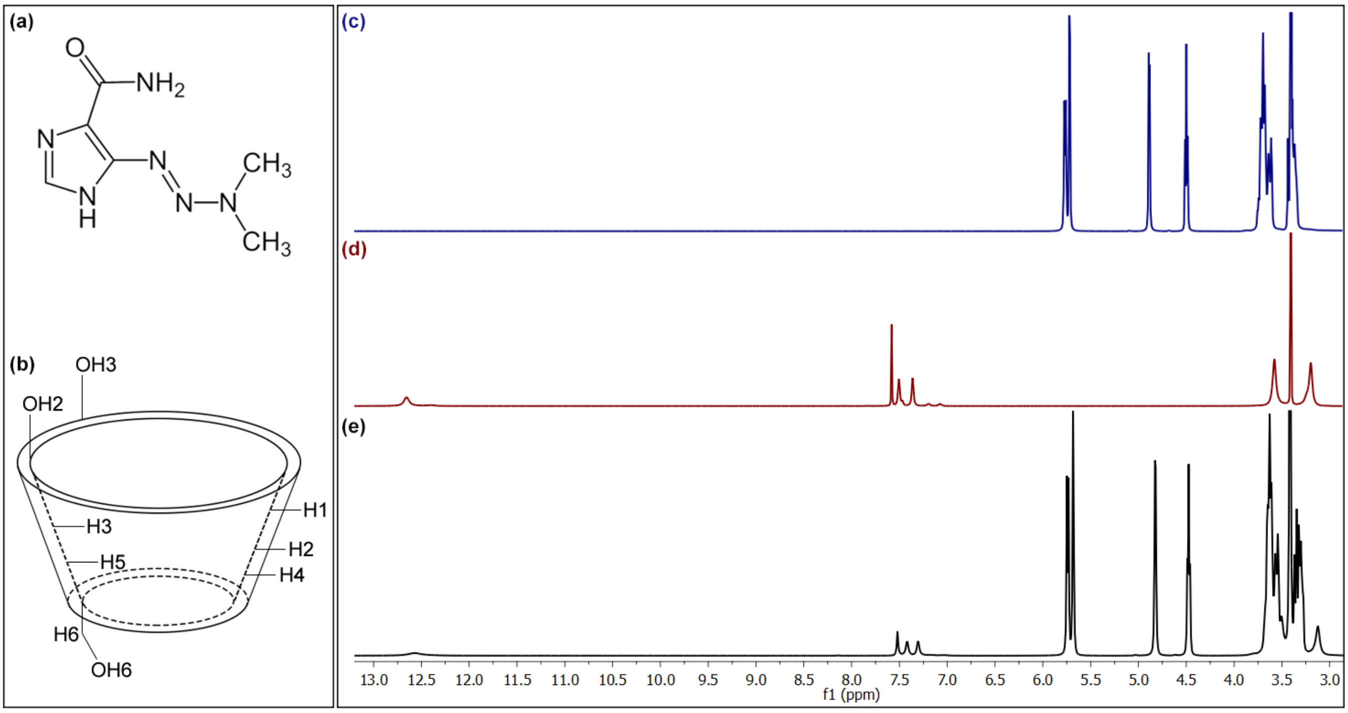

Figure S3. (**a**) Molecular structure of dacarbazine in its two tautomeric forms; (**b**) Cone-type model of β-cyclodextrin indicating the orientation of its protons, which are represented for a unit of glucose. ^{1}H-NMR spectra of: (**c**) β-cyclodextrin; (**d**) dacarbazine; and (**e**) β-cyclodextrin@dacarbazine complex, obtained in DMSO-d_6.

S3. Stoichiometric ratio of β-cyclodextrin@dacarbazine complex

The stoichiometric ratio was calculated by comparing the integration of the proton signals β-cyclodextrin (H1) and dacarbazine (CH_3 and NH) in the ^{1}H-NMR spectra of the β-cyclodextrin@dacarbazine complex (see figure S4). Values are shown in table S1.

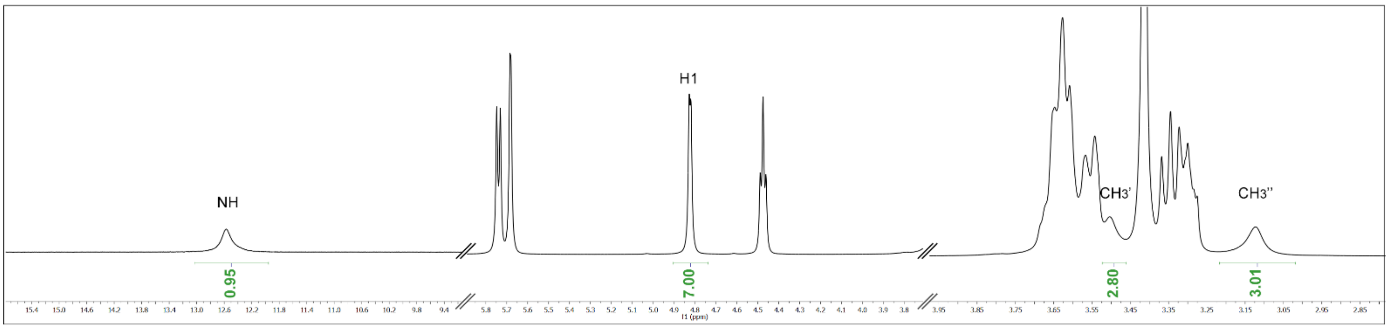

Figure S4. Integrated signals of dacarbazine and β-cyclodextrin protons in the ^{1}H-NMR spectrum of the β-cyclodextrin@dacarbazine complex.

Table S2. Values of the integrated signals of dacarbazine and β-cyclodextrin using the ^{1}H-NMR spectrum of the complex formed.

**Integration of dacarbazine protons**			**Integration of β-cyclodextrin proton**
CH_3'	CH_3''	NH	(reference used)
2.80	3.01	0.95	H1/∫ = 7

S4. Two-dimensional NMR spectrum and docking

Figure S5 shows the full ROESY spectra of the β-cyclodextrin@dacarbazine complex.

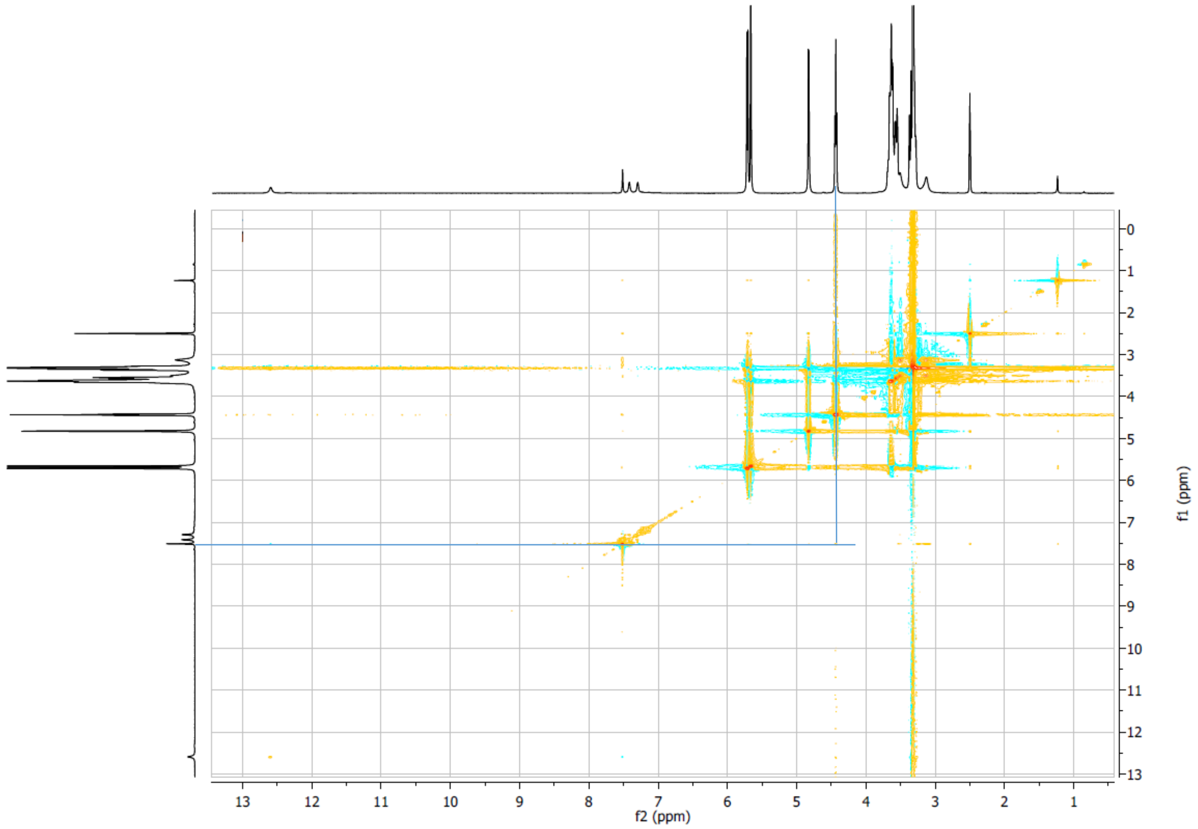

Figure S5. ROESY spectra of the β-cyclodextrin@dacarbazine complex.

S5. Pharmacological parameters of the complex

Figure S6 shows the relation between absorbance at 237 nm of DB solution versus its concentration. The slope corresponds to nanomolar absorptivity coefficient (ε). Figure S7 shows the relation between the concentrations of dacarbazine solubilized versus the concentrations of β-cyclodextrin. The slope corresponds to degree of solubilization.

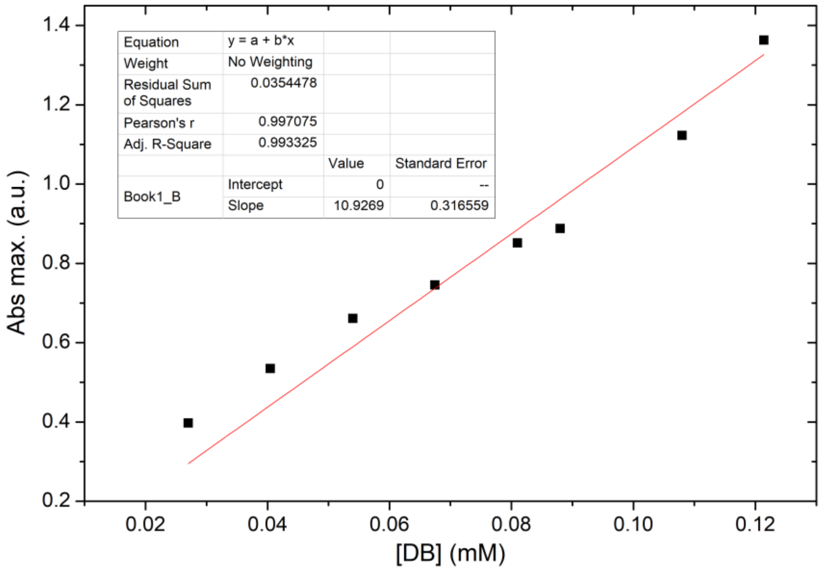

Figure S6. Graph of maximum absorbance at 237 nm versus dacarbazine concentration.

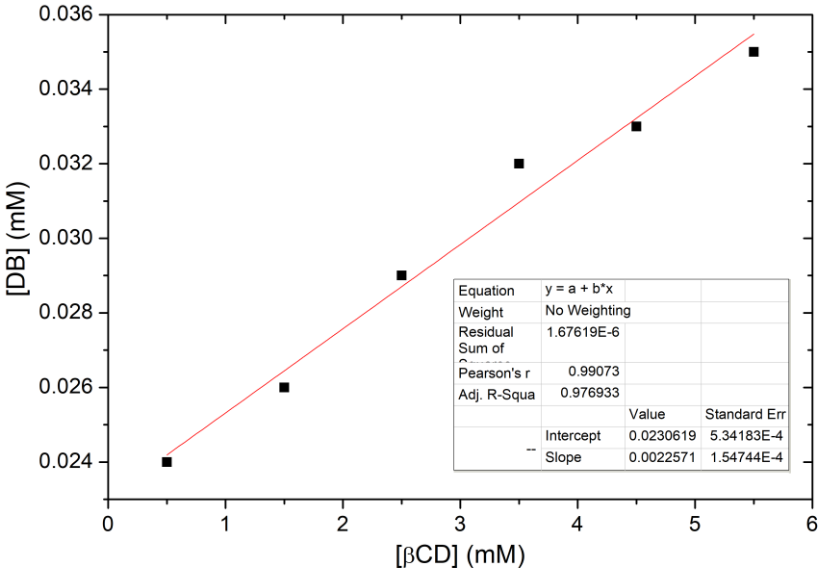

Figure S7. Graph of concentrations of: solubilized dacarbazine versu β-cyclodextrin used.

S6. Characterization of gold nanoparticles

Figure S8 shows the hydrodynamic diameter distribution by intensity of gold nanoparticles stabilized using: citrate and PEG-β-cyclodextrin@dacarbazine, obtained by dynamic light scattering.

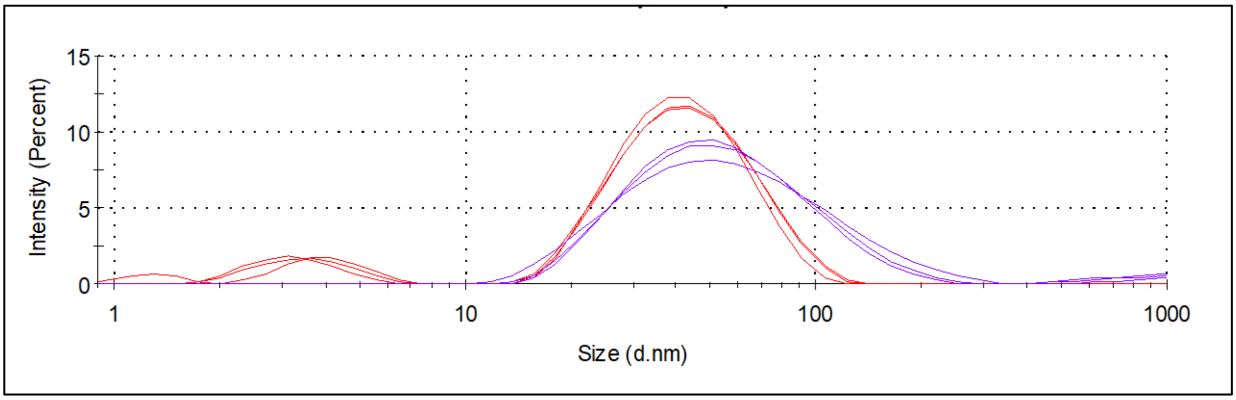

Figure S8. Hydrodynamic diameter distribution by intensity of gold nanoparticles stabilized with: citrate (red) and PEG-β-cyclodextrin@dacarbazine (purple).

Figure S9 shows the micrograph of gold nanoparticles as they were synthesized. Next, Figure S10 and S11 show the size histograms of gold nanoparticles stabilized using: citrate and PEG-β-cyclodextrin@dacarbazine,

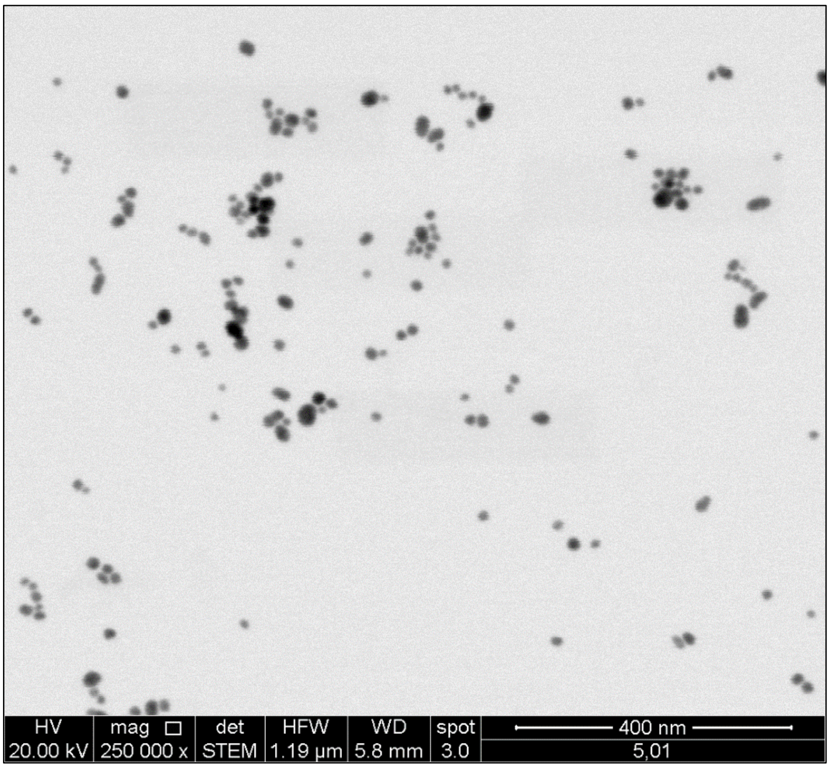

Figure S9. TEM of gold nanoparticles stabilized with citrate.

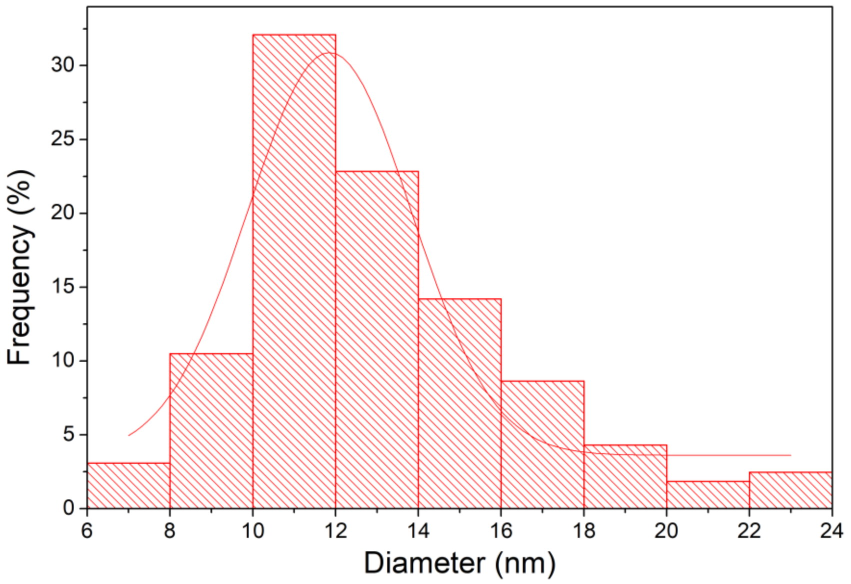

Figure S10. Size histogram of gold nanoparticles stabilized with citrate.

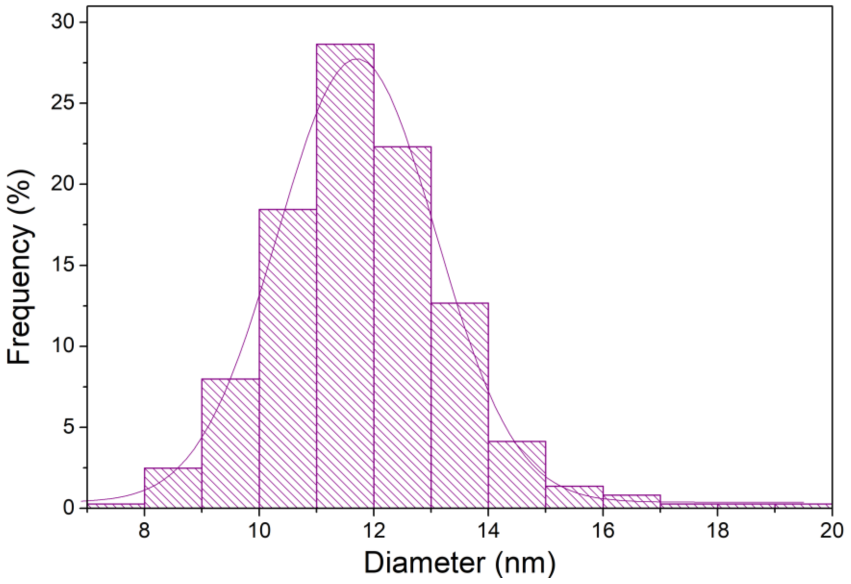

Figure S11. Size histogram of gold nanoparticles stabilized with β-cyclodextrin@dacarbazine and PEG.

Table S3. Mass of dacarbazine in the supernatant, amount of dacarbazine loaded on gold nanoparticles, and efficiency of dacarbazine included in β-cyclodextrin and loaded on gold nanoparticles

n	Dacarbazine in supernatant (µg)	Dacarbazine loaded in gold nanoparticles (µg)	Loading efficiency (%)
1	0.053	40.957	99.9
2	0.028	40.982	99.9
3	0.011	40.999	100.0
4	0.071	40.939	99.8
5	0.070	40.940	99.8
6	0.087	40.923	99.8
Average	0.053 ± 0.029	40.957 ± 0.029	99.9 ± 0.1

S7. Studies using Raman and IR spectroscopy

Table S3 shows the assignment of the vibrational modes corresponding to the signals of the dacarbazine spectrum. Figure S12 shows the FT-IR spectra of dacarbazine, β-cyclodextrin and the β-cyclodextrin@dacarbazine complex.

Table S4. Theorical Raman shift, Raman shift observed in the dacarbazine spectrum and the corresponding vibrational mode.

Theorical Raman Shift (cm^{-1})	Raman shift observed in dacarbazine (cm^{-1})	Assignment
312	330	N-C-C and N-N-C scissoring modes
414	436	N-C-O scissoring
568	558	N-N-N scissoring and amide bond in-plane bending
602	638	N-C-O scissoring and C-N(azide) in-plane bending
	694	
799	802	C-N-C stretching and ring in-plane deformation
800		C-N-C stretching and ring in-plane deformation
912	908	Ring deformation and N-CH_3 stretching
955	964	Ring in-plane deformation
1074	1074	NH_2 rocking and ring in-plane deformation
1100	1117	NH_2 rocking and CH_3 rocking
1152	1137	CH_3 twisting
1233	1182	Aromatic C-N stretching and NH_2 rocking
	1222	
1260	1267	Aromatic C-H bending and ring deformation
1328	1302	C-N-C symmetric stretching
1348	1341	Ring in-plane bending, azide N-N stretching, and C-NH_2 stretching
1387	1372	Ring in-plane bending and azide N-N stretching
1429	1402	CH3 twisting, azide N-N stretching and ring bending
	1411	
1452	1444	CH_3 scissoring
1498	1489	Azide N-N stretching and CH_3 scissoring
1525	1508	Ring stretching and CH_3 scissoring
1526	1545	CH_3 scissoring
1587	1585	NH_2 scissoring
1607	1650	NH_2 scissoring and aromatic C-C stretching
1740		C=O stretching
2998	2920	CH_3 symmetric stretching
3020		CH_3 stretching
3074		CH_3 stretching
3097		CH asymmetric stretching
3139		CH asymmetric stretching
3142		CH asymmetric stretching
3240	3144	Aromatic CH stretching
3285		NH_2 symmetric stretching
3649		Aromatic NH stretching
3727		NH_2 asymmetric stretching

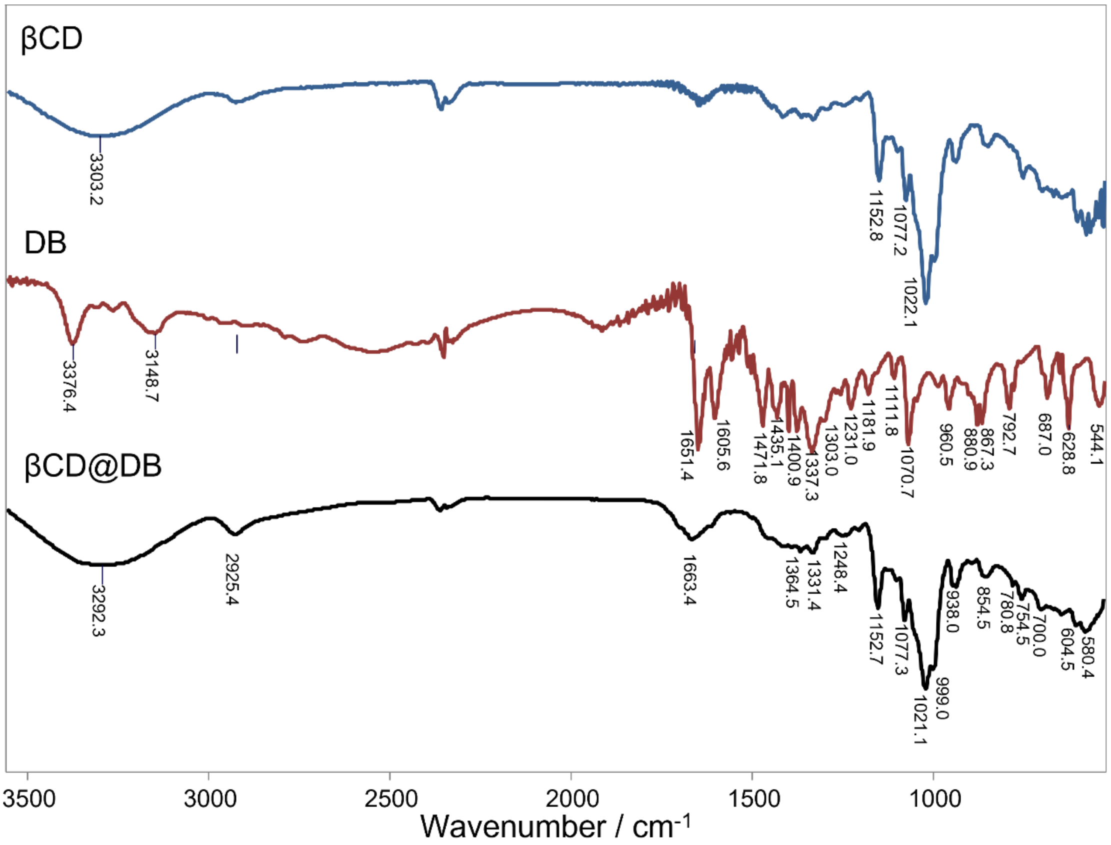

Figure S12 FT-IR spectra of β-cyclodextrin, dacarbazine, and the β-cyclodextrin@dacarbazine complex.

S8. Laser irradiation assays

Table S4 shows the mass released of dacarbazine taken from the organic phase at each time interval, and finally the sum of all mass released, referred to as the "total" mass. The mass of dacarbazine loaded into the nanosystem was 39.0 μg.

Table S5. Mass released of dacarbazine at each time interval, sum of all mass released, and percentage of average cumulant released mass.

Time	Mass (μg)	Mass (μg)	Mass (μg)	Average mass (μg)	Average cumulative mass (%)
15	2.63	4.14	3.51	3.4 ± 0.8	8.8 ± 1.9
30	3.86	4.72	3.57	4.1 ± 0.6	19.2 ± 1.5
45	3.97	5.51	3.70	4.4 ± 1.0	30.4 ± 2.5
60	4.01	6.71	3.82	4.8 ± 1.6	42.9 ± 4.1
**Total released**	**14.5**	**21.1**	**14.6**	**16.7 ± 3.9**	**42.9 ± 4.1**
